# P05.29. Does yoga improve smoking cessation outcomes? A systematic review of the literature

**DOI:** 10.1186/1472-6882-12-S1-P389

**Published:** 2012-06-12

**Authors:** L Carim Todd, S Mitchell, B Oken

**Affiliations:** 1 Oregon Health & Science University, Portland, USA

## Purpose

To evaluate the effectiveness of a yoga intervention for smoking cessation.

## Methods

A systematic search, review and synthesis of existing literature on yoga interventions for smoking cessation was conducted. Online literature searches through MEDLINE, PsycINFO, EBM, PubMed, clinicaltrials.gov and NIH RePORTER were carried out using an array of search terms and combinations. Manual search of reference lists and specific authors was also performed. Studies were selected that had: (1) smoking-related primary outcomes and, (2) an intervention consisting of yoga or a component of yoga (e.g. pranayama).

## Results

Four studies met our inclusion criteria. The variation between studies was substantial in terms of study population, study design, sample size, control condition, type of yoga intervention, implementation of the intervention, adherence rates, length of follow-up and number of outcomes. However, despite the variability and limited number of reports available, data suggests that the practice of yoga might influence the desire and motivation to quit smoking, reduce smoking urges, reduce temptations to smoke, increase pulmonary health awareness and reduce inflammatory response in stressful situations.

## Conclusion

There is some suggestion that yoga could aid in smoking cessation. All four studies found changes in smoking behavior or attitude towards smoking after the intervention. However, the variety of study designs, the non-standardized nature of the interventions, lack of follow-up, and differences in study population and sample size, limit our capacity to draw definitive conclusions. Therefore, in order to accurately assess whether yoga can be an effective component of smoking cessation treatments, there is a strong need for randomized controlled clinical trials with larger sample sizes, clearly defined yoga interventions, longer follow ups, and efficient measures of compliance and adherence.

